# Supplementary material for: Real-time surveillance of international SARS-CoV-2 prevalence using systematic traveller arrival screening: An observational study
Source: PLoS Med. 2023 Sep 8;20(9):e1004283. doi: 10.1371/journal.pmed.1004283 (PMC10516411; doi:10.1371/journal.pmed.1004283)
Supplement: S1 Text — (DOCX) [file pmed.1004283.s001.docx]

**Supplementary Materials: Real-time surveillance of international SARS-CoV-2 prevalence using systematic traveller arrival screening: a retrospective study**

***Calculation of testing probabilities for prevalence estimation***

As described in the main text, we can estimate departure prevalence from arrival testing data as follows. If N individuals are tested at arrival, then the number who test positive is binomially distributed with probability *q*, where:

*q* = P(test positive at arrival | tested negative at departure)

= P(arrival+ | departure–).

We can rewrite this probability in terms of arrival and departure tests:

P(arrival+ | departure–) = P(arrival+ and departure–)/ P(departure–)

If w is the probability an individual is infected at departure (i.e. could in theory test positive), then:

P(arrival+ and departure–) = *w* P(arrival+ and departure– | infected)

P(departure–) = *w* P(departure– | infected) + (1–*w*)

Details of how these probabilities can be calculated are provided in the supplement.

Combining the above probabilities gives the following expression for the probability an individual tests positive at arrival:

$$q= \frac{w P(arrival+ and departure- | infected)}{w P(departure- | infected) + (1-w)}$$

To estimate P(departure– | infected), i.e. the probability of detection at departure given infection, we used the median estimate of testing PCR positive from a cohort study of self-tested healthcare workers [1], taking the first 30 days post-infection in our analysis as the probability was negligible after this point. We assume PCR positivity post-infection for the wildtype variant is representative of positivity for subsequent variants [2,3]. Analytical specificity for PCR tests (i.e. probability of avoid a false positive) is typically very high, estimated to be above 99.9% in UK community testing data [4].

As pre-departure PCR testing had to be within 72 hours of travel, in our baseline scenario we assumed travellers performed a PCR test 2 days before departure, with 1 day travel time to French Polynesia (i.e. 3 days between test and arrival). We assume that the probability of transmission during transit is negligible, given all travellers will have had a negative test at departure, and so the probability of infection post-arrival, given domestic prevalence in French Polynesia was generally low relative to prevalence at departure.

We assumed stable epidemic incidence at departure (i.e. the number of new infections constant over time) and no infections post arrival (i.e. infection prevalence much lower at arrival destination versus departing country). Let *p_i_* be the probability that an infected individual will test positive on day *i* after infection and D be the maximum number of days post-infection considered (D=30 in our analysis, based on the positivity curve in Fig 1D). We therefore have:

$$P(departure- | infected)= \frac{1}{D+1}\sum_{i=0}^{D} {(1-p}_{i})$$

To calculate P(arrival+ and departure– | infected), we first define the probability that a departing infected individual would be *i* days post-infection and go undetected:

$$P\left( depature- \right| i days post infection)= u_{i}= \begin{matrix} 1/D \text{ if } i \leq t_{\text{pre}} \\ {(1-p}_{i-t_{\text{pre}}})/D \text{ if }i>t_{\text{pre}} \end{matrix}$$

We then define the probability that an arriving infected individual would be *i* days post-infection and test positive at arrival, assuming that no infection occurred post-arrival while waiting to perform the test:

$${P\left( arrival+ \right| i days post infection)= a}_{i}= \begin{matrix} 0 \text{if} i\leq t_{post} \\ p_{i}u_{i-t_{\text{post}}} if i>t_{post} \end{matrix}$$

The sum of these probabilities, with index from day 0 to D, gives us the probability an infected individual will test negative at departure and positive at arrival:

$$P(arrival+ and departure- | infected)= \frac{1}{D+1}\sum_{i=0}^{D} a_{i}$$

***Posterior distribution of departure prevalence***

We can calculate the likelihood of observing a given number of positives at arrival using the following expression:

$$P\left( X \right| w)= f(X , N, q) =P(X | N, \frac{w P(arrival+ and departure- | infected)}{w P(departure- | infected) + (1-w)} )$$

where f(X, N, *q*) is the probability mass function of the binomial distribution with size N and probability *q*.

If N is the set of arrival tests, and X is the corresponding number of positives, then we can then use Bayes Theorem to define the posterior probability for being infected at departure, based on the likelihood function for *w* and the prior for *w*:

$$P\left( w \right| X)= \frac{P\left( X \right| w) P(w)}{P(X)}=\frac{P\left( X \right| w) P(w)}{\int_{0}^{1} P\left( X \right| w)P(w)dw}$$

Here we set the prior P(*w*) to the uniform distribution, to reflect an assumption that incoming prevalence may be unknown. Because we only have on parameter to consider, we estimate P(X) numerically. We can then use this posterior probability to estimate the distribution of prevalence (i.e. probability of testing positive) at departure:

$$P\left( \mathrm{prevalence} \right| X )=P\left( w \right| X) P(departure+| infected)= P(w | X)\sum_{i=0}^{D} p_{i}$$

In the text, we report the maximum a posteriori probability (MAP) and the highest posterior density interval (HDPI) for prevalence based on the posterior. We do not report p-values in the manuscript because we were not performing a test of a null hypothesis in a frequentist framework.

***Full travel testing model***

Here we define a general process model for departure and arrival testing, and expected number of individuals who would test positive. This is used to generate illustrative dynamics in Fig 2. Let *p_i_* be the probability that an infected individual will test positive on day *i* after infection and *x_j_* be the number of individuals infected on day *j*. Then the number of infected travellers who would be detected in a departure test on day *k* is given by:

$$\sum_{i=0}^{D} {x_{k-i} p}_{i}$$

Where D is the maximum number of days post-infection considered (D=30 in our analysis). If pre-departure tests are conducted *t_pre_* days before arrival, then the expected number of infected travellers who would go undetected and arrive on day

*k + t_pre_* is given by:

$$\sum_{i=0}^{D} {x_{k-i} (1-p}_{i})+\sum_{i=1}^{t_{pre}} x_{k+i}$$

If post-arrival tests are conducted *t_post_* days after arrival, then the expected number of infected arriving travellers who would test positive on day *k + t_pre_ + t_post_* post-infection is given by:

$$\sum_{i=0}^{D} {x_{k-i} {(1-p}_{i}) p}_{i+t_{pre}+ t_{post}}+\sum_{i=1}^{t_{pre}} x_{k+i}p_{k+i+t_{post}}$$

*In Figs 2 and S1, we simulated test positivity at arrival by generating binomial random samples based on the above probabilities, under different assumptions about the dynamics of x_j_ over time. We also explored potential mis-specification of assumptions about the testing process and timing, as well as variation in sample size, in S1 Fig.*

***Cumulative incidence model***

To estimate departure incidence from estimated prevalence, we used a generalised additive model (GAM) [5] using a Gaussian family, with weekly observations weighted by the variance of our posterior estimates of prevalence. We used a generalised additive model, rather just than bootstrap sampling each weekly prevalence estimate independently, to reflect the fact that observed weekly tests are independent observations from a correlated generative process (i.e. the underlying epidemic). We estimated pre and post 1^st^ May 2021 separately, as arrival testing shifted from day 4 to day 0 at this point, and hence the scaling factor also changed, and GAM prevalence estimates were truncated at 0.

To convert infection prevalence into an estimate of incidence, we divided weekly prevalence by the total density of positivity $\sum_{i=0}^{D} p_{i}$ = 8.6 days. We also calculated the mean duration of shedding $\sum_{i=0}^{D} i p_{i}$ = 9.2 days, and assumed this corresponded to incident positivity 9 days earlier. These values were derived from the cohort study of self-tested healthcare workers described above. This approximation is further explored in S2 Fig.

We generated a bootstrap 95% prediction interval for cumulative incidence by simulating 1000 times from the fitted GAM using observed weekly intervals and testing numbers, calculating cumulative incidence, then estimating the median and central 95% values over time. To compare with seroprevalence data, we assumed a 17 day delay to seroconversion [6]. Cumulative incidence estimated from repeat PCR prevalence has previously been shown to provide a close match to seroprevalence over time in the UK ONS community infection survey [7].

***Alternative departure protocol***

In our baseline scenario we assumed all travellers performed PCR tests within 2 days of departure. If we instead assume that all travellers performed antigen tests at departure within 1 day of travel, which they were eligible to do after May 2021, we estimated 3.6% (95% CrI: 3, 4.1%) and 3.3% (95% CrI: 2.7, 3.9%) during the BA.1 waves in France and the USA respectively (S1 Fig). These values are lower than the estimates for PCR testing at departure because antigen tests would detect fewer departing infections on average, and hence we would expect prevalence measured at arrival to be closer to the true departure prevalence.

***Reconstruction of cumulative incidence***

In our baseline scenario, we reconstruct cumulative incidence in Fig 4 by scaling prevalence by the mean duration of positivity. To explore the performance of this approximation, we simulated daily incidence (S2A Fig), converted this incidence to prevalence by convolving with probability of PCR positivity post-infection (S2B Fig). We then re-estimated incidence from these prevalence data using our baseline scaling approximation (red line in S2C Fig) as well as deconvolution based on the Moore-Penrose generalized inverse (blue line in S2C Fig). Estimated cumulative incidence is shown in S2D Fig, showing that the simple scaling approximation gives a reasonable reconstruction of cumulative incidence for plausible prevalence dynamics.

**References**

1. Hellewell J, Russell TW, The SAFER Investigators and Field Study Team, The Crick COVID-19 Consortium, CMMID COVID-19 working group, Beale R, et al. Estimating the effectiveness of routine asymptomatic PCR testing at different frequencies for the detection of SARS-CoV-2 infections. BMC Med. 2021 Dec;19(1):106.
2. Bouton TC, Atarere J, Turcinovic J, Seitz S, Sher-Jan C, Gilbert M, et al. Viral dynamics of Omicron and Delta SARS-CoV-2 variants with implications for timing of release from isolation: a longitudinal cohort study. MedRxiv. 2022.
3. Hay JA, Kissler SM, Fauver JR, Mack C, Tai CG, Samant RM, et al. Quantifying the impact of immune history and variant on SARS-CoV-2 viral kinetics and infection rebound: A retrospective cohort study. eLife. 2022 Nov 16;11:e81849.
4. Coronavirus (COVID-19) Infection Survey: methods and further information. Office for National Statistics. 2023. Available from: <https://www.ons.gov.uk/peoplepopulationandcommunity/healthandsocialcare/conditionsanddiseases/methodologies/covid19infectionsurveypilotmethodsandfurtherinformation#test-sensitivity-and-specificity> Accessed 1 Dec 2022
5. Wood SN. Generalized Additive Models: An Introduction with R. CRC Press. 2006.
6. Borremans B, Gamble A, Prager K, Helman SK, McClain AM, Cox C, et al. Quantifying antibody kinetics and RNA detection during early-phase SARS-CoV-2 infection by time since symptom onset. eLife. 2020 Sep 7;9:e60122.
7. Abbott S, Funk S. Estimating epidemiological quantities from repeated cross-sectional prevalence measurements. MedRxiv. 2022.
