# Supplementary figures and images for: Real-time surveillance of international SARS-CoV-2 prevalence using systematic traveller arrival screening: An observational study
Source: PLoS Med. 2023 Sep 8;20(9):e1004283. doi: 10.1371/journal.pmed.1004283 (PMC10516411; doi:10.1371/journal.pmed.1004283)

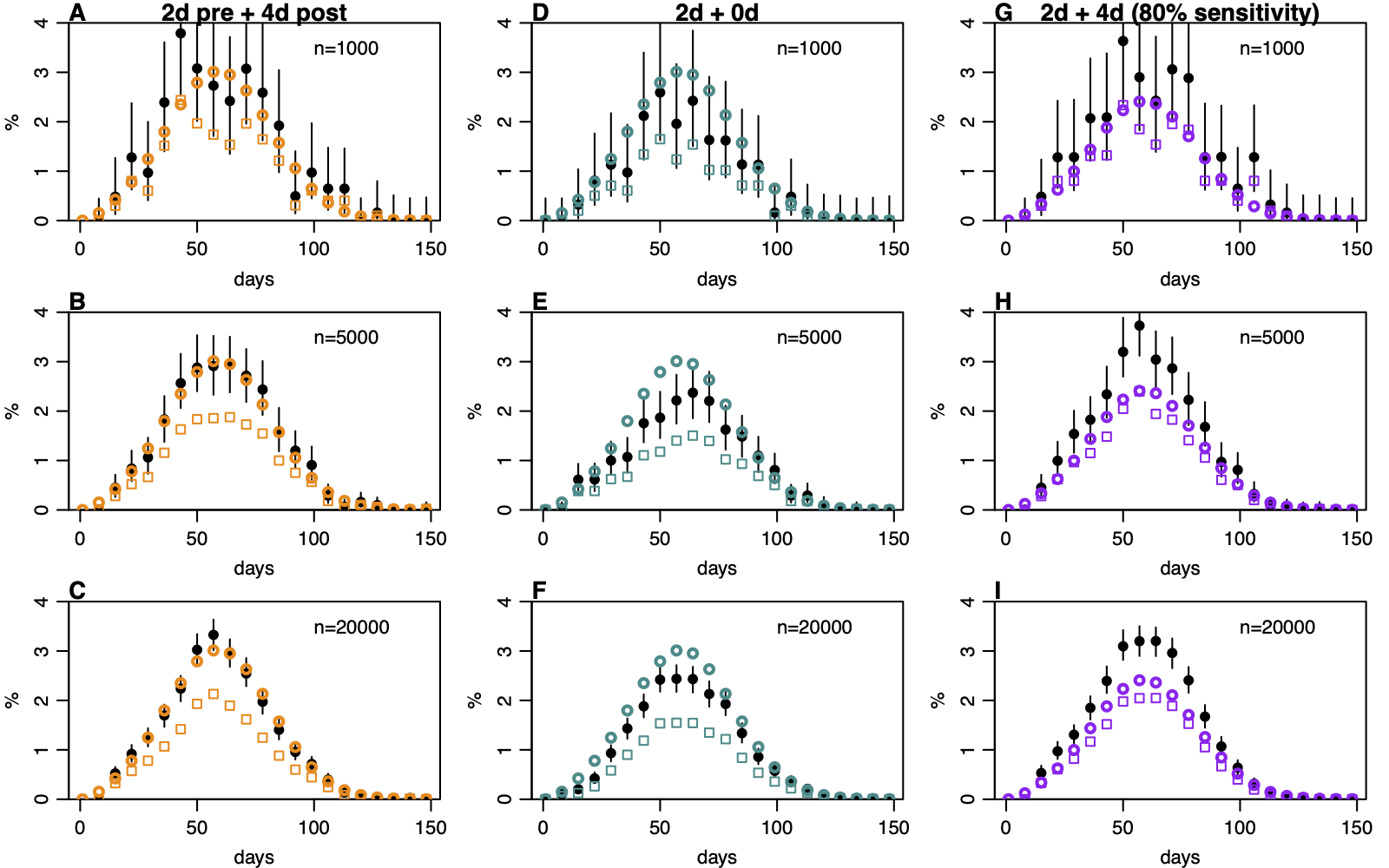

Supplement: S1 Fig — (A) Reconstruction of simulated epidemics from arrival testing data, assuming 100 arrivals tested per week. Solid points, “true” prevalence at departure with lines showing 95% binomial confidence interval; squares, measured prevalence at arrival in simulated scenario with a test 2 days before departure with 1 day in transit and another test 4 days post-arrival (i.e., 2d + 4d), with circles showing reconstructed departure prevalence from these data. (B) Same as (A), but with 5,000 arrivals tested per week. (C) Same as (A), but with 20,000 arrivals tested per week. (D) Reconstruction of simulated epidemics from arrival testing data when test timing is mis-specified (shown in green). In the simulation, there is a test 2 days before departure with 1 day in transit and another test on day of arrival. However, inference is performed with a test 2 days before departure with 1 day in transit and another test 4 days post-arrival. (E) Same as (D), but with 5,000 tested per week. (F) Same as (D), but with 20,000 tested per week. (G) Reconstruction of simulated epidemics from arrival testing data when test sensitivity is mis-specified (shown in purple). Simulated tests have peak sensitivity equal to 80% of the peak in our baseline analysis, with inference performed under assumption sensitivity is unchanged. Otherwise, the scenario is same as in (A). (H) Same as (G), but with 5,000 tested per week. (I) Same as (G), but with 20,000 tested per week. (TIFF) [file pmed.1004283.s002.tiff]

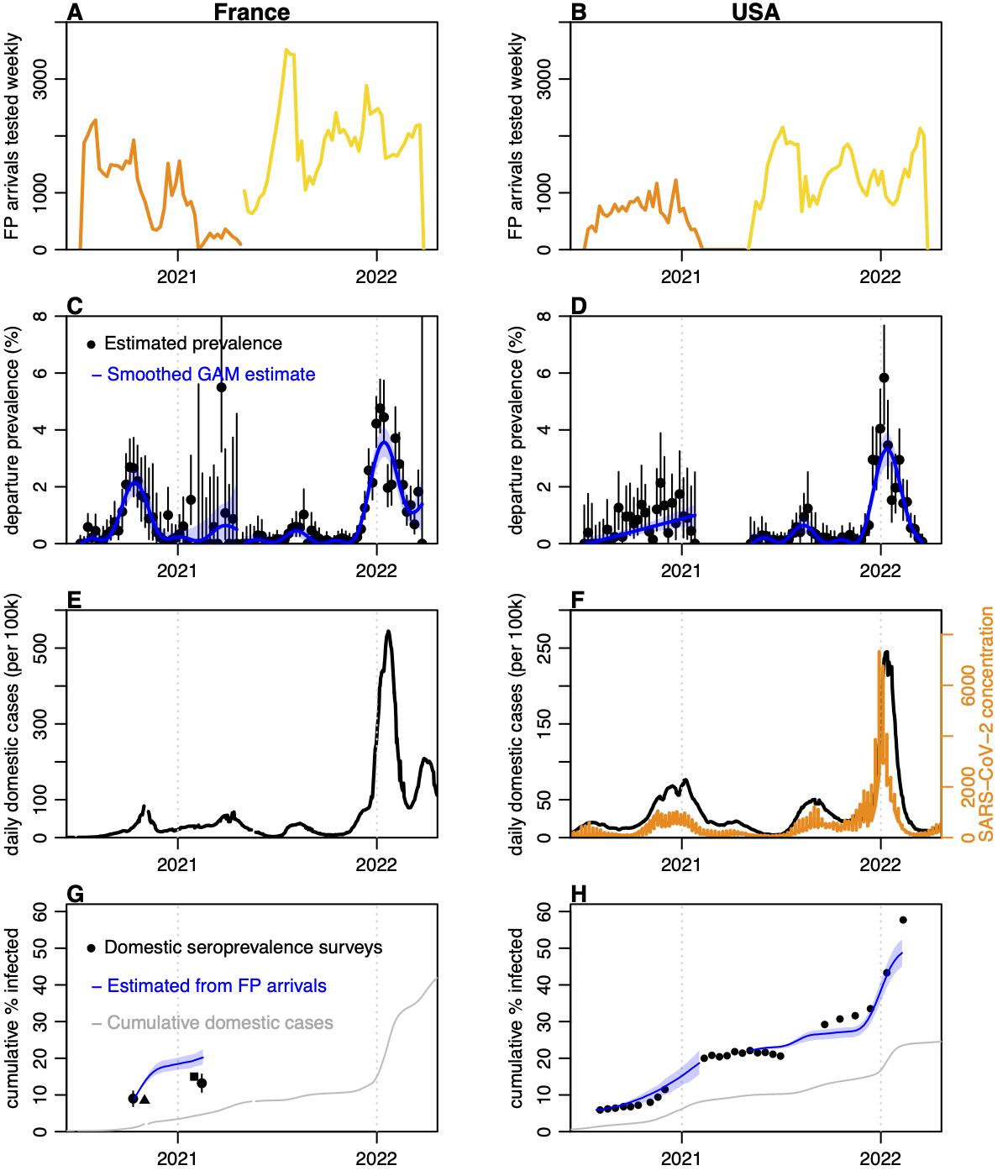

Supplement: S2 Fig — Panels otherwise as described in main text (Fig 4). (TIFF) [file pmed.1004283.s003.tiff]

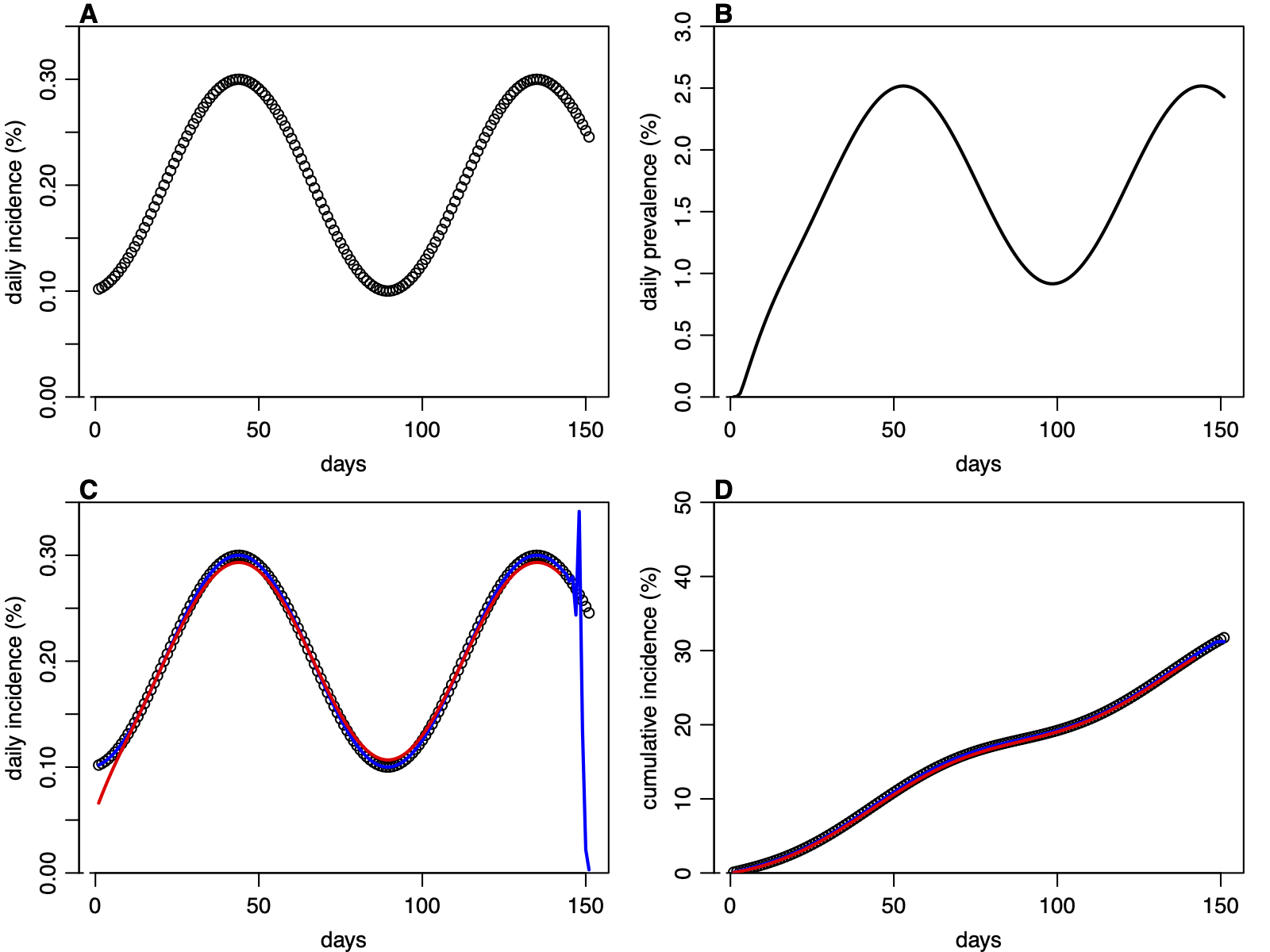

Supplement: S3 Fig — (A) Simulated daily incidence. (B) Simulated daily prevalence based on the convolution of incidence in (A) and median probability of PCR positivity in Fig 1B. (C) Reconstruction of incidence from prevalence in (B) using our baseline scaling approximation (red line) as well as deconvolution using the Moore–Penrose generalized inverse (blue line, showing numerical instability at end of time series). (D) Comparison of simulated cumulative incidence (black dots), with estimated cumulative incidence using our baseline scaling approximation (red line) as well as deconvolution using the Moore–Penrose generalized inverse (blue line). (TIFF) [file pmed.1004283.s004.tiff]
