# Supplementary material for: Real-time surveillance of international SARS-CoV-2 prevalence using systematic traveller arrival screening: An observational study
Source: PLoS Med. 2023 Sep 8;20(9):e1004283. doi: 10.1371/journal.pmed.1004283 (PMC10516411; doi:10.1371/journal.pmed.1004283)
Supplement: Attachment — Submitted filename: Response_to_reviewers_V2_1.docx [file pmed.1004283.s005.docx]

-----------------------------------------------------------

Requests from the editors:

GENERAL

Please respond to all editor and reviewer comments detailed below in full.

Please include line (and page) numbers starting at 1 and in continuous sequence thereafter.

COMMENTS FROM THE ACADEMIC EDITOR

Thanks for your submission and congratulations on proposing a potentially unique case for traveller screening data. Please find below some requests:

(1) Most countries are using environmental surveillance (i.e. wastewater surveillance) as a leading indicator. Would suggest including that triangulation and comparison as part of this submission, if feasible.

We agree that this would be a useful comparison, and have added data from the US as a comparison to Figure 4, alongside a mention in the Discussion.

(2) Please elaborate on utility of these data for other countries and the larger public health community (e.g. would you propose the UK used these data as a proxy for other countries' transmission levels?).

We now elaborate on the value of such data in the Discussion, as well as the limitations of the approaches commonly used during COVID-19 in their absence:

“In the UK, studies such as REACT-1 and ONS have provided continuity in local understanding of community infections, but such data streams have been extremely rare globally, given their expense and logistical complexity. However, our analysis shows that routine traveller testing can enable similar insights at an international scale, using protocols that were already being implemented in many countries, offering a substantial improvement on commonly used estimation methods based on reported cases, hospitalisations or deaths”

(3) Please elaborate on potential selection biases involved with using traveller data. A couple examples include that these travellers may only represent the wealthy from US/France rather than travellers across socioeconomic strata and also misses symptomatic individuals.

We now elaborate on potential biases in the Discussion:

“Although our estimates reflected national-level patterns of seroprevalence in the US and France, the age distribution of travellers may be different to the age distribution of the population at departure, particularly if there are additional constraints on unvaccinated individuals such as children travelling, as occurred from mid-2021 until early 2022. Moreover, travellers may be from wealthier or less-risk averse groups, who may have a different exposure risk to the wider population. Demographic insights could be expanded in future epidemics by routinely reporting data on epidemiologically relevant values such as age and occupation, which would allow adjustment for potential biases when estimating prevalence in countries of departure.”

We also discuss potential representative issues when comparing serosurveillance from France with our estimates:

“The volume of travel from France declined substantially during that wave, which may have affected representativeness of travellers, as risk-averse individuals could have been less likely to travel, and risk-taking individuals more likely to be infected in transit.”

We also elaborate on why our estimates are unlikely to be biased by symptom status:

“Although symptomatic individuals may have avoided travel, test positivity also tends to peak around the time of symptom onset [Hay et al, eLife, 2022], and hence these individuals would have been detected at departure had they travelled, a process that is already accounted for in the analysis presented here.”

(4) For the French and US analyses, could you contextualize with serosurveillance and case surveillance data from those countries rather than the UK (the results cites UK infection surveys after the analyses which was confusing)?

We now show a comparison of case data, serosurveillance and our estimates from arrival data in Figure 4, as well as moving the UK infection survey contextual data to the discussion.

(5) There is a lot going on in the figures. I really wanted to hone in on the triangulation of travellers data to nationally representative seroprevalence data. This appears to be in C and D? Are the readers supposed to take away that the trend is the same using both data sources? Furthermore, for E and F are these simply data from other studies? It is not immediately clear how these travellers data are included in those panels

We agree that it would be helpful to simplify and clarify the figures, and now label Figure 4 more clearly, as well as adding wastewater data for comparison.

TITLE

Please revise your title according to PLOS Medicine's style. Your title must be nondeclarative and not a question. It should begin with main concept if possible. "Effect of" should be used only if causality can be inferred, i.e., for an RCT. Please place the study design ("A randomized controlled trial," "A retrospective study," "A modelling study," etc.) in the subtitle (ie, after a colon).

We have made this edit.

ABSTRACT

Abstract Background:

Please ensure that the final sentence clearly states the study question.

We have rephrased this sentence to make the study question clearer.

Abstract Methods and Findings:

Please ensure that all numbers presented in the abstract are present and identical to numbers presented in the main manuscript text.

Please define ‘PCR’ at first use for the reader

We have made these edits.

Please provide brief demographic details of the study population (e.g. sex, age, ethnicity, etc)

To protect passenger privacy, these details were not linked to test results. We have provided a description of this in the Methods, and elaborated on linkage issues in the Discussion.

Please include/clearly define the number of participants, length of follow up, and main outcome measures.

Please define the numerical values contained within parentheses

Please quantify the main results with 95% CIs and p values. When reporting p values, please report as p<0.001 and where higher the exact p value as p=0.002, for example. If not reporting p values, please clearly state the reasons why not, to help facilitate transparent data reporting.*

We define the number of participants tested and the outcomes of interest (i.e. PCR test result) in the methods section. We have also amended parentheses to be clear what is being shown, and clarify in the supplementary methods why we do not include p-values: “We do not report p-values in the text because we were not performing a test of a null hypothesis.”

When reporting 95% CIs suggest the use of commas to separate upper and lower bounds as opposed to hyphens as these can be confused with reporting of negative values.*

*Please check and amend throughout the main manuscript, tables, figures and supporting files where relevant.

Please include any important dependent variables that are adjusted for in the analyses.

Please include numerators and denominators used to derive percentages.

In the last sentence of the Abstract Methods and Findings section, please detail 2-3 primary limitations of the study's methodology.

We have made these changes. International prevalence percentage estimates shown in Figure 4 were estimated in a statistical analysis (see Methods and Supplement), so there is not a simple denominator to report. However, volume of testing and positives overall is shown in Figure 3.

Abstract Conclusions:

Please emphasize what is new and address the implications of your study and in doing so please avoid assertions of primacy (‘We report for the first time...’), ‘In this study, we observed...’ may be useful.

Please interpret the study based on the results presented in the abstract, emphasizing what is new without overstating your conclusions.

Please address the study implications without overreaching what can be concluded from the data

Please avoid vague statements such as ‘these results have major implications for policy/clinical care’. Mention only specific implications substantiated by the results.

We have edited the conclusion to make the implications clearer.

AUTHOR SUMMARY

At this stage, we ask that you include a short, non-technical Author Summary of your research to make findings accessible to a wide audience that includes both scientists and non-scientists. The authors summary should consist of 2-3 succinct bullet points under each of the following headings:

• Why Was This Study Done? Authors should reflect on what was known about the topic before the research was published and why the research was needed.

• What Did the Researchers Do and Find? Authors should briefly describe the study design that was used and the study’s major findings. Do include the headline numbers from the study, such as the sample size and key findings.

• What Do These Findings Mean? Authors should reflect on the new knowledge generated by the research and the implications for practice, research, policy, or public health. Authors should also consider how the interpretation of the study’s findings may be affected by the study limitations. In the final bullet point of ‘What Do These Findings Mean?’, please describe the main limitations of the study in non-technical language.

The Author Summary should immediately follow the Abstract in your revised manuscript. This text is subject to editorial change and should be distinct from the scientific abstract. Please see our author guidelines for more information: [https://journals.plos.org/plosmedicine/s/revising-your-manuscript#loc-author-summary](https://protect-eu.mimecast.com/s/Nmw3C7XRKi2QVL2s4fBgO?domain=journals.plos.org)

This has been added.

INTRODUCTION

Please indicate whether your study is novel and how you determined that.

If there has been a systematic review of the evidence related to your study (or you have conducted one), please refer to and reference that review and indicate whether it supports the need for your study.

We have amended the Introduction to indicate the novelty of our analysis:

“Although some countries with strict restrictions on traveller numbers have reported the number of arriving infections detected over time [11] and data have been reported from brief or small-scale testing programmes [12–14], there has been no systematic long-term data published from global travel testing.”

Please define ‘ONS’ and ‘REACT-1’

We now define these acronyms.

METHODS and RESULTS

We ask all authors of modelling studies to ensure the inclusion of specific items, derived from Geoffrey P Garnett, Simon Cousens, Timothy B Hallett, Richard Steketee, Neff Walker. Mathematical models in the evaluation of health programmes. (2011) Lancet DOI:10.1016/S0140-6736(10)61505-X. Please review the list below and ensure that all items are included in the relevant parts of the main manuscript:

The above paper focuses on modelling of health interventions (i.e. exploring future scenarios with a priori parameters), whereas we perform a statistical analysis of observed data, adjusting for how test positivity changes post-infection. However, we have updated the manuscript to include the below points where relevant.

* Please provide a diagram that shows the model structure, including how the disease natural history is represented, the process and determinants of disease acquisition, and how the putative intervention could affect the system.

A schematic of the testing process is included in Figure 1A–D

* Please provide a complete list of model parameters, including clear and precise descriptions of [the meaning of each parameter, together with the values or ranges for each, with justification or the primary source cited, and important caveats about the use of these values noted].

Model parameters – specifically, the probabilities underlying the likelihood calculation – are now detailed in the supplement.

* For uncertainty analyses, please state the sources of uncertainties quantified and not quantified [can include parameter, data, and model structure].

We now outline the uncertainty quantification, via the binomial likelihood and posterior estimation, in the updated Supplement. We also specify the parameters (e.g. median estimated PCR positivity from healthcare worker data.

* Please provide sensitivity analyses to identify which parameter values are most important in the model. Uncertainty estimates seek to derive a range of credible results on the basis of an exploration of the range of reasonable parameter values. The choice of method should be presented and justified.

Sensitivity analysis to the testing process is provided in the appendix (e.g. whether PCR or antigen test used).

* Please discuss the scientific rationale for this choice of model structure and identify points where this choice could influence conclusions drawn. Please also describe the strength of the scientific basis underlying the key model assumptions.

We outline the rationale for the binomial likelihood function in the Methods, and the accompanying testing process in the Supplement.

Methods/’Data’ – this section both defines and discusses the background to the methodology. Please ensure that the methods section serves to only clearly define the methodology used and restrict background information/discussions to the relevant sections of your manuscript. Please revise accordingly. Please define PCR at first use.

The Methods section covers the methods used to generate the data and the methods used to analyse them, so we believe this content sits better in this section than in the background. We now define PCR at first use.

Methods/’travel testing model’ – ‘D=30 days’ what does the D depict here, please define for the reader.

This has been defined.

Ethics statement – please define LSHTM for the reader.

This has been defined.

As above, PLOS Medicine requests that main results are quantified with 95% CIs and p values. When reporting p values, please report as p<0.001 and where higher the exact p value as p=0.002, for example. If not reporting p values, please clearly state the reasons why not, to help facilitate transparent data reporting.

The main results (i.e. adjusted prevalence estimates) are now more clearly reported with 95% CIs. We now clarify in the supplementary methods why we do not include p-values: “We do not report p-values in the text because we were not performing a test of a null hypothesis.”

When reporting 95% CIs suggest the use of commas to separate upper and lower bounds as opposed to hyphens as these can be confused with reporting of negative values. Please check and amend throughout the main manuscript, tables, figures and supporting files where relevant.

We have made these edits.

Results, para 5 – please clearly define the numerical values contained within parentheses for the reader

We now clarify these are 95% credible intervals (as we are now generating posterior estimates), with 95% binomial confidence intervals shown in Figure 3.

FIGURES & SUPPORTING FIGURES

Please consider avoiding the use of green and/or red to improve accessibility of your figures to those with colour blindness.

Please ensure that all figure captions clearly describe the content of the figure without the need to refer to the text.

Please ensure that all abbreviations are defined in the figure captions for the reader, including those used to report statistical information.

We have now run our figures through colour blindness simulators. Figure 1 is clearly labelled so is accessible. We have now added labels to Figure 2 to improve accessibility here as well, and checked captions for clarity.

Figure 1 – you report credible intervals (CrI) and elsewhere confidence intervals (CI) should it be one or the other? Please clarify/revise as necessary.

Figure shows outputs from a previous Bayesian analysis, so CrI is correct here. We then use the median probability estimate from this analysis to adjust seroprevalence in a binomial model in the remainder of the manuscript. We have updated the Figure 1 caption to make this clearer.

Figure 3 – please define HCW, PCR, Ct, WT

We have added these definitions

Figure 5 – please clearly define the meaning of the dots and lines as well as the grey dotted line.

We have added definitions.

DISCUSSION

Please present and organize the Discussion as follows (avoiding the use of sub-headings): a short, clear summary of the article's findings; what the study adds to existing research and where and why the results may differ from previous research; strengths and limitations of the study; implications and next steps for research, clinical practice, and/or public policy; one-paragraph conclusion.

We have re-structured the Discussion to follow this order.

REFERENCES

Please ensure that in the bibliography, up to but no more than 6 author names are listed followed by et al., in the event that more than 6 individuals contribute to an individual study. Please ensure that journal name abbreviations are those found in the National Center for Biotechnology Information (NCBI) databases. Please see our website for other reference guidelines [https://journals.plos.org/plosmedicine/s/submission-guidelines#loc-references](https://protect-eu.mimecast.com/s/xTkYC8EVLUWzP9WSVIVF8?domain=journals.plos.org)

We have made these changes

Comments from the reviewers:

Reviewer #1: Kucharski et al. analyse COVID testing data on persons arriving in French Polynesia, and make useful inferences on infection prevalence in other parts of the world.

Overall comments

This is a very nice and careful analysis of a complex dataset. There are some limitations, and one general question I would have is about the public health value of this data to the people paying for this data (possibly the taxpayers in French Polynesia?), since it relates to infection prevalence in other locations. Nevertheless, countries should be willing to donate resources for the greater international good. In addition, I don't think it is too plausible that COVID policies in France or the United States would be based on surveillance data on travelers into French Polynesia. France and the United States should be capable of collecting data on infection prevalence in their own locations. Notwithstanding these issues, there is clearly incredible scientific value of the data, which were generated anyway because of on-arrival testing of all arrivals. I agree with authors that a global network of sentinel locations collecting this type of data would be a fantastic resource to

allow inferences on infection prevalence globally, and WHO should certainly consider this in the next pandemic. However for surveillance purposes, the ideal data would be on-arrival testing and reporting of pre-departure tests, because the shape of the "hole" in Figure 1D is absolutely critical to all of authors' inferences.

We agree that the main potential of such surveillance in future would be through a global network of sentinel locations that could enable triangulation, as hopefully came across in our discussion. The public health value of travelling testing data for French Polynesia was two fold: 1) trying to limit virus dissemination (once identified travellers found positive for SARS-CoV-2 were asked to self-isolate and were not allowed to travel to outer islands); 2) get early information on the introduction of new variants circulating in other countries and be prepared to local circulation (strengthening of vaccination campaigns and communication on shielding measures). The cost of traveller surveillance in French Polynesia was initially covered by the local government (until July 2021), then by the travellers themselves when registering on the (mandatory) travellers identification platform. The use of travellers surveillance data from French Polynesia to describe the infection prevalence in other locations is therefore a secondary use of such data on top of its primary aims. We have updated the methods to include this background.

Perhaps worthy of mention is that the travel measures in French Polynesia between February and August 2021 was part of a successful effort to keep COVID out of the community (correct, from Figure 3C?) by combining it with on-arrival quarantine as well as other control measures? Perhaps the paper could be expanded with a brief mention of this, because obviously on-arrival COVID testing will only affect COVID control if combined with (1) minimization in daily arrivals and (2) strict management of those that do arrive and (3) domestic outbreak control measures. Without these other three components it is unlikely that arrival testing will have any impact.

We now mention this point in the Results:

“This illustrates the value of delayed arrival testing during rising SARS-CoV-2 transmission. However, given the percentages undetected, travel testing and quarantine would still need to be combined with other measures, such as a reduction in traveller numbers or domestic control measures, to prevent sustained transmission.”

One other final comment/concern is the complexity of methodological description used for presentation to a general audience. Authors might consider illustrating the methods with a flow diagram or schematic to show the concepts involved, with formulas left to the appendix.

We agree that the methods could be made more accessible. We now present the methods in terms of overall probabilities relating to the schematic in Figure 1, with the details given in the appendix.

Major comments

1. Have authors assumed that PCR sensitivity in French Polynesia is equal to PCR sensitivity in the pre-departure tests (mainly in France and the US)? I would question that assumption. PCR sensitivity can vary substantially between laboratories. Figure 1D has a chunk out of it assuming that pre-departure tests have a particular sensitivity, but authors have basically no information on that. Authors also assume constant PCR sensitivity over time for different variants which is somewhat unlikely.

Our baseline assumption is that sensitivity is the same at departure and arrival. We therefore now include a sensitivity analysis in the Supplement showing how much estimates using our methodology would vary if tests used were in reality less sensitive than the assumed PCR positivity curve. As expected, this leads to an underestimation of prevalence. However, the degree of situational awareness about the proportion infected from such an estimate would still be much closer to true infection dynamics than domestic symptom-based reporting systems such as case testing data. The similarity between our estimates and observed seroprevalence, particularly in the US, suggests any bias is unlikely to be large, but these issues could be explored more in future with better linkage on tests at departure, as we now cover in the Discussion:

“Linkage with test results at departure would also enable validation of assumptions about PCR sensitivity at each stage of travel.”

Bouton et al (cited in the submitted manuscript) found no substantial difference in cultural positivity or viral decay by variant or vaccination status. Other studies (e.g. Hay et al, eLife, 2022) also since found that individual variation in positivity – which is accounted for in our binomial likelihood – typically dominates over variant or immune status. However, if sensitivity was to vary substantially by variant, it would also be an issue for interpretation of other infection surveys and wastewater studies. We now cover this in the discussion:

“We also assume that PCR positivity over time for the wildtype variant is representative of subsequent variants, based on similarities observed in cohort studies [24]. If positivity were to vary substantially, then this would need to be accounted for with different adjustments based on dominant variants in countries of departure. If viral shedding profiles were very different by variant, similar adjustments would also need to be made when inferring infection dynamics from other data sources, such as community infection surveys or wastewater data.”

2. In third paragraph of results, at the end, there is an argument about the proportion of infections that would be picked up by delayed testing but the point of this analysis is unclear. Other studies have already shown that unless imported infections are reduced to an absolute trickle, COVID will spread in the community (unless the community is already in lockdown). Delayed on-arrival COVID testing may or may not have an advantage over immediate on-arrival testing but there is a far bigger story to look at there, than just the proportion of infections picked up. This paper seems to be about the inferences that can be drawn on prevalence in France and the US from the arrival testing, not about the value of the arrival testing for the control of COVID in French Polynesia (see comments above).

We included this analysis for context, given the primary objective of the delayed arrival testing was to detect infections, rather than obtain unbiased estimates of departure prevalence. It was notable that delayed testing was useful for both of these objectives, as we now mention in the Results:

“This illustrates the value of delayed arrival testing for detecting infections during rising SARS-CoV-2 transmission, as well as for reconstructing departure prevalence.”

3. End of first paragraph of results "Our approach could therefore generate reliable estimates for simulated departure prevalence dynamics…" that's good news that your model can recover input parameters in a simulation, but reliability mainly depends on sample size, accuracy would be a more important metric to mention here, particularly in simulations where the pre-departure PCR sensitivity is misspecified in your model. At a guess, the inference on trends in prevalence would be more accurate than the inference on exact prevalence values at a given time, provided that pre-departure PCR sensitivity remains fairly constant over time even if misspecified in the model?

We realise it would be helpful to be clearer about uncertainty and bias in our simulation recovery analysis. We now simulate random numbers of positive travellers based on simulated incidence, then randomly detect infected departing passengers based on PCR test positivity, then randomly detect infected passengers at arrival, before using these values to recover the original prevalence. The updated results are shown in Figure 2, with a sensitivity analysis, including mis-specification of the testing protocol and test sensitivity, in the Supplement.

Although trends in prevalence would be less sensitive to assumptions about test sensitivity, there is substantial value in having an estimate of the magnitude of infection, rather than just changes in growth, which we now mention in the Discussion:

“In turn, these measurements of infection dynamics could be used in estimation of situation awareness metrics such as the reproduction number, as well as estimation of population-level epidemic dynamics for scenario models, where knowledge of the extent of infection can substantially improve statistical inference”

4. For Figure 4, how did you know where travelers came from? Was this just based on where the inbound flight had taken off from, or was it based on some type of health declaration? Some passengers may have connected onto the inbound flights and actually come from other locations? Ref 23 seems to be referring to tourists but many arriving persons would be returning residents? And ref 23 may not survive in the future, it might be better to extract the raw data you used from that website and include it as an appendix file.

We have expanded the methods to detail traveller origin. In brief, origin was based on the airport of departure, under the assumption that returning residents were also likely to have been infected in the country of departure:

“To protect privacy, age, nationality and address of tested passengers were not available for analysis. The origin of tested passengers was therefore defined based on the flight number. Between 15th July 2020 and 17th November 2021, passengers on flights from Paris, France had a direct transit (via Canada or Guadeloupe) so it was not possible to leave or join the plane in transit. Later, this transit was via the USA (Los Angeles), where it was possible to leave or join the plane mid-journey. Between July 2020 and March 2022, there were 464,728 incoming travellers: 229,744 tourists and 234,984 returning residents [22]. We do not know whether passengers that took their plane in Paris or in Los Angeles came originally from another country, but because 90% of tourist arrivals in 2021 were reported as from these two original countries on arrival forms, we made the assumption that infected tourists or returning residents reflect infections acquired in the USA and France. ”

Archiving data from ref 23 is a good suggestion – we now include a copy on the repository accompanying this manuscript, and will deposit a stable DOI version of the code/data (e.g. on Zenodod) alongside the final version of manuscript.

5. The end of the fourth paragraph provides UK data for context but that would seem to be better suited to the Discussion than the Results section. I don't think you have any directly comparable data on prevalence in France or the US for the studied periods, which means there is not really a way to validate your results. That does reveal the potential importance of your inferences - if valid - to reveal something that otherwise is not known. But it would be preferable if you had at least some data for France or the United States to triangulate. Comparison of your PCR results with serology is not particularly convincing in this case, unless you can show perhaps how ONS PCR data closely match with ONS seropositivity as well?

We agree that the UK comparison would be better placed in the Discussion and have now moved this. There is no similar prevalence data for France and US, which as you suggest shows the value of such travel testing data. Because we are comparing PCR testing with seroprevalence, we have also added a citation with comparison of PCR and ONS seropositivity:

“Cumulative incidence estimated from repeat PCR prevalence has previously been shown to provide a close match to seroprevalence over time in the UK ONS community infection survey [Abbott et al, 2022].”

Reviewer #2: In this paper, the authors describe results from a SARS-CoV-2 traveler testing program run in French Polynesia between July 2020 and March 2022. As the authors highlight, the use of traveler testing data can potentially provide a less biased estimate of local infection prevalence, serve as a leading indicator for changes in prevalence, and also contain information on the prevalence of countries where travelers originate from. The authors demonstrate an approach for analyzing arrival/departure testing data, accounting for potential biases associated with epidemic stage, e.g., rising, falling steady, and when an individual was tested wrt to travel, e.g., how far in advance of travel or how long after arriving. Additionally, they provide estimates of SARS-CoV-2 prevalence in French Polynesia, France, and the United States. Given the recent attention associated with airport testing, the work here is quite timely. However, I do have a few questions/comments, which I

hope the authors find constructive.

1. Do we know how infection rates among travelers is related to infection rates in the entire population? Given that there are socio-economic biases between travelers and non-travelers and socio-economic biases in infection rates, it seems as though some correction is needed because travelers are not an iid sample from the entire population.

There are no directly comparable estimates of infection rates in France and the US because community infection surveys (like ONS and REACT-1 in the UK) were not conducted. We therefore included a comparison of cumulative infections in France and the US estimated from our data with changes in domestic seroprevalence in these countries, finding similar patterns over time, particularly in the US. We cover this comparison in the Methods and the Supplement, noting the consistency between seroprevalence and community infection dynamics in the UK:

“Cumulative incidence estimated from repeat PCR prevalence has previously been shown to provide a close match to seroprevalence over time in the UK ONS community infection survey”

We also discuss representativeness in Discussion:

“Although our estimates reflected national-level patterns of seroprevalence in the US and France, the age distribution of travellers may be different to the age distribution of the population at departure, particularly if there are additional constraints on unvaccinated individuals such as children travelling, as occurred from mid-2021 until early 2022. Moreover, travellers may be from wealthier or less-risk averse groups, who may have a different exposure risk to the wider population. Demographic insights could be expanded in future epidemics by routinely reporting data on epidemiologically relevant values such as age and occupation, which would allow adjustment for potential biases when estimating prevalence in countries of departure.”

2. Additionally, do we know if travelers are more generally at risk of getting infected? You might imagine that the distribution of social contacts for someone who is a traveler is higher than for someone that isn't, so, all else equal, their risk of getting infected would be higher.

The relative risk to travellers was likely to be context specific, depending on the stage of the pandemic. Travellers may be less risk averse (increasing risk) but conversely, they may also be wealthier (meaning they are in an occupation with less exposure to others during COVID). We address these potential issues in the updated Discussion:

“Although our estimates reflected national-level patterns of seroprevalence in the US and France, the age distribution of travellers may be different to the age distribution of the population at departure, particularly if there are additional constraints on unvaccinated individuals such as children travelling, as occurred from mid-2021 until early 2022. Moreover, travellers may be from wealthier or less-risk averse groups, who may have a different exposure risk to the wider population. Demographic insights could be expanded in future epidemics by routinely reporting data on epidemiologically relevant values such as age and occupation, which would allow adjustment for potential biases when estimating prevalence in countries of departure.”

We also discuss potential representative issues when comparing serosurveillance from France with our estimates:

“The volume of travel from France declined substantially during that wave, which may have affected representativeness of travellers, as risk-averse individuals could have been less likely to travel, and risk-taking individuals more likely to be infected in transit.”

3. Could either points 1 or 2 above be related to why you find that traveler positivity is a leading indicator of country-wide prevalence? I could also imagine that simply having a less biased estimate would provide a leading indicator as well.

The main reason for positivity acting as a leading indicator is likely to be the biological and epidemiological lags involved in cases and hospitalisations, because it takes time for people to develop (severe) symptoms, seek a test and obtain the result. We now mention this in the Discussion:

“Commonly cited international COVID-19 indicators such as cases or hospitalisations are strongly dependent on symptom severity and domestic access to testing, as well as being delayed outcomes relative to infection incidence.”

4. Related to the above points, how do your estimates of prevalence compare to hospitalizations, test positivity, and wastewater positivity (to the extent it's available) in the various countries? Given that we expect the relationship between true prevalence and test positivity to shift during the pandemic, I'd expect to see a similar shift in the relationship between test positivity and your estimate of prevalence from travel testing.

We were not able to find routinely reported wastewater estimates for France, but we have added data on wastewater in US for comparison. We also include cases for comparison, alongside seroprevalence. We do not include hospitalisations/deaths as these generally lag cases. Test positivity among symptomatic cases depends on domestic testing protocol and test-seeking behaviour, so we do not believe this is as useful a comparator to include as seroprevalence or wastewater, which provide a closer measure of cumulative incidence and current prevalence respectively.

5. Many individuals also believe that wastewater surveillance can provide an unbiased, real-time estimate of prevalence. I would encourage the authors to add a brief discussion of how wastewater surveillance and traveler testing might complement each other. For example, wastewater surveillance also tends to be a leading indicator, but that's almost certainly due to biases in clinical testing wrt to timing of infection. As a result, I could imagine that traveler testing might prove to be a true leading indicator.

We agree this would be useful to include, and now include US data in Figure 4, as well as mentioning in the Discussion:

“Such estimates could also be triangulated against other potential leading indicators, including viral concentrations in wastewater, although interpretation of wastewater data would rely on additional analysis to convert into a comparable measure of the proportion of the population who are currently infected.”

6. In the "Prevalence Model" section, the authors provide an estimate of the mean duration of positivity (8.6 days). Was this number obtain empirically? If so, it's not clear how it estimated. If it's not an empirical estimate, where do you obtain this value?

We now clarify in the supplement that positivity was based on published estimates from a self-tested health-care worker cohort.

7. Related to the above, is there data on whether infection duration varied across variants? The reported serial interval for Omicron is between 2 and 4 days (although Abbott et al. report a generation time of 1.5 - 3.2 days, which would meant the serial interval would likely be narrower).

Abbott, S., Sherratt, K., Gerstung, M., & Funk, S. (2022). Estimation of the test to test distribution as a proxy for generation interval distribution for the Omicron variant in England. medRxiv.

Kim, D., Ali, S. T., Kim, S., Jo, J., Lim, J. S., Lee, S., & Ryu, S. (2022). Estimation of serial interval and reproduction number to quantify the transmissibility of SARS-CoV-2 omicron variant in South Korea. Viruses, 14(3), 533.

Ito, K., Piantham, C., & Nishiura, H. (2022). Estimating relative generation times and relative reproduction numbers of Omicron BA. 1 and BA. 2 with respect to Delta in Denmark. medRxiv.

Bouton et al (cited in the submitted manuscript) found no substantial difference in cultural positivity or viral decay by variant or vaccination status. More detailed cohort studies have also shown only small differences across variants (Russell et al, MedRxiv 2023). Other studies (e.g. Hay et al, eLife, 2022) also found that individual variation in positivity – which is accounted for in our binomial likelihood – typically dominates over variant or immune status. We now mention this in the discussion:

“We also assume that PCR positivity over time for the wildtype variant is representative of subsequent variants, based on similarities observed in cohort studies [24]. If positivity were to vary substantially, then this would need to be accounted for with different adjustments based on dominant variants in countries of departure. If viral shedding profiles were very different by variant, similar adjustments would also need to be made when inferring infection dynamics from other data sources, such as community infection surveys or wastewater data.”

The serial interval over time can be highly dependent on interventions (Ali et al, Science, 2020), as so we believe this is less reliable as an indicator of variant-specific infection duration.

Reviewer #3:

This paper is concerned with a potentially useful method for surveillance of covid prevalence using traveller screening data. While the idea and development are interesting, there is a large number of methodological issues with the paper. Specific comments are given below.

The authors will need to be scientifically honest about the quantification of uncertainty of the proposed approach. At a somewhat simplistic level they suggest that their data, which contain information from about a thousand cases, can reconstruct and accurately estimate the epidemic dynamics of the USA and France. If this method is to be used in practice one would need a precise estimate of the associated uncertainty at the very least.

This task also involves estimating the uncertainty of functions of probabilities and explicit discussions on using the bootstrap or the delta method should be given.

We agree it would be useful to dig further into the uncertainty associated with estimation from travel testing. In Figure 3 of the submitted paper, we presented arrival testing in terms of binomial uncertainty. In the updated Methods and Supplement, we now define the distribution of arrival prevalence in full, i.e. binomially distributed with a probability that can be decomposed into conditional probability dependent on the proportion infected at departure. This makes it possible to define a likelihood function and hence infer departure prevalence from the number tested at arrival and number positive at arrival. Full details are provided in the supplement.

For clarity, the information contained within our dataset is more than just the number of positives – we’re focusing on prevalence, so what matters is the ratio between the number of people tested and the number positive. For example, if 100,000 people were tested and zero were positive, that would provide a large amount of information about prevalence.

In addition to the variance of those probabilities, potential bias issues should be discussed in detail, especially since some of the probabilities involved are small in which case the associated MLEs/sample proportions can be highly unstable. For such cases, methods like the Firth correction or Bayesian approaches give more accurate estimates (in terms of mean square error) and could be considered in this paper.

To improve generalisability of the methods, we now use the likelihood function defined for arrival tests to generate a posterior probability for departure prevalence. In our analysis, we use the uniform distribution as a prior, but in future, others could use this approach to incorporate existing information if available (e.g. from domestic studies of prevalence). In Figure 4, we now present the maximum a posteriori probability (MAP) and the highest posterior density interval (HDPI) for prevalence as our estimates, using a GAM weighted by variance of weekly posterior estimates to show the trend in prevalence.

In addition to the technical details of the bias and variance properties of the estimates used, there is a subtle notion related to the aim of the paper. Are the travellers used for screening a truly representative sample of the population at the country of origin? And if not does this really matter?

It would be desirable to disentangle the two and offer a fair account of what the paper does and does not achieve with the proposed approach.

A fundamental challenge for COVID surveillance has been understanding population infection dynamics from available testing data. In the absence of a very small number of studies with community testing regardless of symptoms (e.g. ONS and REACT-1), countries have typically relied on symptomatic case testing and healthcare admissions, both of which are unrepresentative for an infection that has such as strong severity gradient with age. We now mention this in the Discussion:

“Commonly cited international COVID-19 indicators such as cases or hospitalisations are strongly dependent on symptom severity and domestic access to testing, as well as being delayed outcomes relative to infection incidence.”

In the Results, we compare our estimates to national-level seroprevalence from France and the US to show that our estimates provide a reasonable proxy for the magnitude of infection, and to case data and wastewater to show that they capture the timing of large epidemic waves. However, we have included some additional discussion of potential representativeness issues, and how these could be investigated further in future:

“Although our estimates reflected national-level patterns of seroprevalence in the US and France, the age distribution of travellers may be different to the age distribution of the population at departure, particularly if there are additional constraints on unvaccinated individuals such as children travelling, as occurred from mid-2021 until early 2022. Moreover, travellers may be from wealthier or less-risk averse groups, who may have a different exposure risk to the wider population. Demographic insights could be expanded in future epidemics by routinely reporting data on epidemiologically relevant values such as age and occupation, which would allow adjustment for potential biases when estimating prevalence in countries of departure.”

Please discuss reliable ways to validate these estimates, including against independent data, possibly from seroprevalence studies. The "bootstrap prediction intervals" discussed at the end of "prevalence model" are in-sample estimates and therefore conservative.

We included a comparison to seroprevalence studies in the previous version of Figure 4, but now expand this with additional US data, which shows that our estimates of cumulative incidence from travel testing data provide

Do the authors account for the effects of false positives/negatives and the associated uncertainty?

The potential for false negatives is explicitly included in the likelihood function via the p_i_ term (i.e. probability an infected individual will test positive), while analytical specificity for PCR is very high (above 99.9%) and so we assume in our analysis that any effect is negligible.

Please discuss the availability of the various data sources, including the cohort study of self-tested healthcare workers.

Novel data sources are available alongside accompanying code on the GitHub repository linked in the Methods section. Additional data sources are available in cited papers and accompanying repositories (e.g. for the healthcare worker analysis in Hellewell et al, BMC Med, 2021).

I think the authors implicitly assume in their calculations that the probability of infection during travel is negligible, please discuss.

That is correct, and we have updated the Supplementary Methods to discuss:

“We assume that the probability of transmission during transit is negligible, given all travellers will have had a negative test at departure, and so it the probability of infection post-arrival, given domestic prevalence in French Polynesia was generally low relative to prevalence at departure.”

Would working with R_t estimates offer an alternative or complementary approach to the proposed technique? In some ways it would settle some of the issues like automatically adjusting for a growing/declining epidemic in the country of origin.

R_t estimation would not necessarily solve the issue of a growing/declining epidemic, as conversion of prevalence data to incidence can still be subject to bias depending on the epidemic phase (Abbott et al, MedRxiv, 2023 – cited in the text). Prevalence estimates, rather than just rates of change such as growth rates of R_t, are also valuable for calibrating scenario models of population dynamics to inform policy, because they provide information on the magnitude as well as the growth of infection. We now mention these applications in the discussion:

“In turn, these measurements of infection dynamics could be used in estimation of situation awareness metrics such as the reproduction number, as well as estimation of population-level epidemic dynamics for scenario models, where knowledge of the extent of infection can substantially improve statistical inference.”

When discussing the results (like the 60/40 contribution in figure 3) it would be useful to try and disentangle the relative importance of assumptions and data. Some of those results heavily depend upon the assumption made so some clarification would help.

Figure 3E–F were forward simulations based on the testing protocol outlined in Figure 3. The figure shows the sensitivity of the results to epidemic growth and test used. We have also expanded the Results to make this clearer:

“Based on protocols implemented, we estimated that arrival PCR testing at day 4 would have been to expected to detect around 60% of infected individuals within the first 10 days of their infection if an epidemic in the departure location was growing at 10% per day (Figure 3E), and around 40% during an epidemic that was declining 10% per day (Figure 3F). This illustrates the value of delayed arrival testing for detecting infections during rising SARS-CoV-2 transmission, as well as for reconstructing departure prevalence. However, given the percentages undetected, travel testing and quarantine would still need to be combined with other measures, such as a reduction in traveller numbers or domestic control measures, to prevent sustained transmission. In reality, the proportion of infections detected would be binomially distributed, and hence also subject to randomness, as well as from any uncertainty in the test sensitivity at departure and arrival.”

A similar idea, of exploring traveller screening data for surveillance is explored in (Bastani et al. Nature 599, p.108-113, 2021) where additional covariate information is utilised. The authors should discuss their work in the context of the relevant literature.

We agree it would be worth including related applications of travel screening. The example of Bastani et al focuses on individual level predictions to optimise test targeting, which is a different objective to generating an unbiased estimate of population-level prevalence. We now cover this work in the introduction:

“Algorithms have also been developed to predict individuals more likely to test at arrival based on demographic characteristics such as age and country of origin (Bastani et al, Nature, 2021), which can be useful for detecting infections, but less relevant if the objectives to obtain unbiased estimates of overall population prevalence.”

Page numbering would be useful in the review process, please add them.

This was an oversight – numbers have now been added.
