# Supplementary material for: Real-time surveillance of international SARS-CoV-2 prevalence using systematic traveller arrival screening: An observational study
Source: PLoS Med. 2023 Sep 8;20(9):e1004283. doi: 10.1371/journal.pmed.1004283 (PMC10516411; doi:10.1371/journal.pmed.1004283)
Supplement: Attachment — Submitted filename: Response_to_reviewers_V3.docx [file pmed.1004283.s006.docx]

GENERAL
Thank you for your detailed and considered responses to previous editor and comments. Please see below for further comments that we require you address prior to publication.

TITLE
Please replace ‘a retrospective…’ with ‘an observational…’

We have made this edit.

AUTHOR SUMMARY
Lines 63 onwards suggest removing the CIs to improve accessibility to the non-scientific reader.

We have made this edit.

Line 72 – this statement is rather vague and doesn’t really tell us anything specific. Suggest removing.

We have made this edit.

METHODS
Line 153 - please define PCR at first use in the main manuscript.

We have made this edit.

FIGURES
If this is not possible to begin axes of graphs at zero, please show a break in the axis.
Figure 4 caption – please define ‘GAM’ for the reader.

We have made these edits.

DISCUSSION
Line 354 – suggest ‘using arrival data from French Polynesia between July 2020 and March 2022’ instead.

We have made this edit.

REFERENCES
Please ensure all web references include an accessed date.

We have added these dates, and also fixed a formatting error on reference 18.

SUPPORTING INFORMATION
Please cite your Supporting Information as outlined here: [https://journals.plos.org/plosmedicine/s/supporting-information](https://protect-eu.mimecast.com/s/hk53CD8vnCXQZzjS8Lx1p?domain=journals.plos.org)

S1 Figure – please define ‘d’ (days) in the caption for the reader, please provide a legend what the different colors and letter refer to.

We have updated the caption accordingly

S2 Figure – please define ‘FP’ in the caption for the reader, please provide a legend what the different colors and letter refer to.

We have updated the caption accordingly. Colours are described in the caption and in figure legends.

Supplement – please ensure the reference format follows our guidance detailed here [https://journals.plos.org/plosmedicine/s/submission-guidelines#loc-references](https://protect-eu.mimecast.com/s/iAB_CExwoHBKj21H4AJCV?domain=journals.plos.org)
Please ensure all web references include an accessed date.

We have updated, in particular adding access dates.
